# Viral surveillance beyond detection: JMTV and the need for ensemble approaches in emerging virus discovery

**DOI:** 10.1128/spectrum.02210-26

**Published:** 2026-09-08

**Authors:** Vibhu Prasad

**Affiliations:** 1 Department of Microbiology and Molecular Medicine, Faculty of Medicine, University of Geneva 28535, Geneva, Switzerland; National Chung Hsing University, Taichung, Taiwan

**Keywords:** emerging viruses, viral surveillance, phylogeny

## Abstract

The recent report by T. Murillo, L. E. Enrique Chaves-González, S. Temmam, S. Bermúdez, et al. (Microbiol Spectr 14:e04078-25, 2026, https://doi.org/10.1128/spectrum.04078-25) expands the known geographic and ecological range of Jingmen tick virus (JMTV) by detecting the virus in *Amblyomma mixtum* ticks collected from horses in Costa Rica. This is an important finding because *A. mixtum* can feed on wildlife, domestic animals, and humans, creating a possible interface for virus movement across various hosts. The study also places the Costa Rican virus in a wider phylogenetic context, linking it to JMTV diversity reported from other regions. However, the detection of viral RNA in ticks should not be interpreted as proof of local disease, human infection, or active transmission, especially in the absence of supporting results. Instead, it reflects an important signal for careful viral surveillance. Here, I discuss how JMTV illustrates the need for ensemble approaches that combine field sampling, phylogeny, segment-level genome analysis, serology, experimental validation, and data-driven virus discovery tools.

## COMMENTARY

In recent years, virus discovery has become increasingly driven by next-generation sequencing, which has significantly changed how new and emerging viruses are found and followed. During the COVID-19 pandemic, SARS-CoV-2 genomic surveillance provided one of the clearest examples of sequence-based discovery occurring in near real time, with epidemiological and phenotypic assessment of viral variants (1, 2). Newly emerging variants of concern were identified while their effects on transmission, immune escape, clinical severity, and public health measures were still being defined (3). In many cases, however, the first sign of a virus is not a disease outbreak, virus isolation, or a clear clinical manifestation but a viral sequence detected in an animal, arthropod, human, or environmental sample (4). Such detection is important, but it also creates a challenge: how should we interpret a new viral genome when little is known about its biology, host range, transmission, or pathogenicity?

Jingmen tick virus (JMTV) is a good example of this challenge. JMTV was first described as a tick-associated segmented RNA virus with an unexpected relationship to flaviviruses (5). Classical flaviviruses, such as dengue virus, Zika virus, and yellow fever virus, have a single positive-sense RNA genome. However, JMTV is different and has a segmented genome, with some segments encoding proteins related to flavivirus replication proteins and other segments encoding more divergent proteins (5). This unusual genome organization made JMTV important not only as a tick virus but also as a reminder that virus diversity can be underappreciated when we search only for familiar genome structures.

The new study by Murillo and colleagues reports the detection and phylogenetic characterization of JMTV in *Amblyomma mixtum* ticks from Costa Rica (6). The authors collected host-feeding ticks from horses and recovered three complete viral genome segments and one partial segment. This is notable because *A. mixtum* is a broadly feeding tick species. It can parasitize wildlife, domestic animals, and humans and therefore sits at an ecological interface where viruses may move between different hosts. The phylogenetic analysis showed that the Costa Rican JMTV sequence is related to other JMTV sequences reported from the Americas and from more distant regions (6). The authors suggest that Jingmenviruses in the Americas may reflect more than one introduction route. They also show that the Costa Rican virus belongs to a broader genetic context that includes sequences from regions where human detections have been reported (6, 7). This makes the finding relevant for public health surveillance, especially because JMTV and related Jingmenviruses have been associated with febrile illness in some studies (7, 8).

However, the interpretation of the newly discovered JMTVs should remain careful, as several facts remain unproven. The detection of viral RNA in a tick does not prove that the tick is an efficient vector. It does not prove that the horse was infected. It does not prove that people in Costa Rica are being infected. It also does not prove that JMTV is causing disease in this region. A feeding tick may contain viral RNA because the tick itself is infected, because the blood meal contains viral RNA, or because of more complex ecological or sampling factors. It is important to distinguish this because virus discovery can move faster than biological interpretation. Once a virus is detected in a medically relevant vector, there is a temptation to describe it as an emerging pathogen. Sometimes this may be correct. But in other cases, detection only marks the beginning of the investigation. For JMTV in Costa Rica, the safest conclusion is not that a new disease has been established, but that a virus with possible medical relevance has been detected in a tick species with human and animal contact. This is already important, but it is different from proving pathogenicity or spread.

For segmented viruses, genome detection is only the first step. Each segment carries its own evolutionary history, and related viruses may exchange genome segments through reassortment. Therefore, a newly detected genome could be a set of linked but potentially different evolutionary signals. This is especially relevant for JMTV, whose segmented genome combines flavivirus-like replication proteins with more divergent structural components (5). In this context, phylogeny becomes more than a classification tool. It helps distinguish shared ancestry, segment reassortment, host or vector movement, and sampling bias.

Earlier comparative work on genome size and gene-content relationships showed, albeit in a different biological setting, that correlations between genomic features can be misleading if phylogenetic structure is ignored (9). Similarly, when new viral genomes are discovered, genome organization, gene content, segment relationships, and phylogenetic placement should be interpreted together rather than independently, especially for viruses with segmented genomes. This is where an ensemble approach becomes valuable. No single method can fully define the importance of a newly detected virus. Field sampling with broad geographical coverage can show where the virus is present and avoid surveillance disparity in sampling efforts and sequencing capacity. Genome sequencing can define its genetic structure. Phylogeny can place it in an evolutionary context. Segment-by-segment comparison can reveal possible reassortment or unusual genome combinations. Serology can show whether vertebrate hosts have been exposed. Culturing the virus in different tick or mammalian cell lines and animal or vector studies can test whether the virus can replicate and be transmitted. Epidemiology can ask whether infection is associated with disease. Hence, each layer answers a specific question.

Computational virus discovery can add another layer to this ensemble. Recent studies have developed data-driven approaches to identify viral hallmark sequences and assemble viral genomes from public sequencing data sets (10). These approaches demonstrate how systematic mining of sequence data can uncover unexpected viral diversity and refine our understanding of viral evolution. Similar strategies could be useful for JMTV and other Jingmenviruses.

Overall, the study by Murillo and colleagues is an important surveillance signal (6). It expands the known distribution of JMTV and identifies *A. mixtum* as a tick species that should be included in future studies. The presence of JMTV RNA in ticks does not yet establish clinical or public health relevance, but it provides a strong reason to apply an ensemble, One Health approach to evaluate potential risk. These could be focused on whether ticks carry replicating virus, whether vertebrate hosts are infected, whether exposed humans develop antibodies, whether infection correlates with symptoms, and whether viral segments show evidence of reassortment or local evolution.

## Supplementary Material

Reviewer comments

## SUPPLEMENTAL MATERIAL

OPEN PEER REVIEW reviewer-comments.pdf An accounting of the reviewer comments and feedback.

ASM does not own the copyrights to Supplemental Material that may be linked to, or accessed through, an article. The authors have granted ASM a non-exclusive, world-wide license to publish the Supplemental Material files. Please contact the corresponding author directly for reuse.
