# Supplementary material for: Viral surveillance beyond detection: JMTV and the need for ensemble approaches in emerging virus discovery
Source: Microbiol Spectr. 2026 Sep 8;14(10):e02210-26. doi: 10.1128/spectrum.02210-26 (PMC13689438; doi:10.1128/spectrum.02210-26)
Supplement: Supplementary file 1 [file reviewer-comments.pdf]

ASM | Microbiology Spectrum

Viral surveillance beyond detection: JMTV and the need for ensemble approaches in emerging virus discovery

Vibhu Prasad

Corresponding Author(s): Vibhu Prasad, Universite de Geneve

**Review Timeline:**	Submission Date:	June 30, 2026
	Editorial Decision:	July 31, 2026
	Revision Received:	July 31, 2026
	Accepted:	August 13, 2026

Editor: Day-Yu Chao

Reviewer(s): The reviewers have opted to remain anonymous.

Transaction Report:

(Note: With the exception of the correction of typographical or spelling errors that could be a source of ambiguity, letters and reports are not edited. The original formatting of letters and referee reports may not be reflected in this compilation.)

DOI: https://doi.org/10.1128/spectrum.02210-26

Re: Spectrum02210-26 (Viral surveillance beyond detection: JMTV and the need for ensemble approaches in emerging virus discovery)

Dear Prof. Vibhu Prasad:

Thank you for the privilege of reviewing your work. Below you will find my comments, instructions from the Spectrum editorial office, and the reviewer comments.

The author provided an in-depth discussion regarding the discovery of JMTV in Costa Rica and suggested an emsemble approach for further investigation on its public health importance. The overall writing is comprehensive with only minor comments

1. Line 24, ...sequencing can be clarified as "next-generation sequencing".
2. Line 82, "Field sampling can show where the virus is present" can be further clarified as "Field sampling with broad geographical coverage can show where the virus is present and avoid surveillance disparity in sampling efforts and sequencing capacity".
3. Line 86, "Cell culture..." can be further clarified as "Virus culture in different tick or mammalian cell lines..."
4. Line 97-101, I suggest consolidating these sentences, such as "The presence of JMTV RNA in ticks is not yet clinically relevant, but it is a strong reason to use the ensemble approach to evaluate the risk to public health and propose the One-Health approach."

Please return the manuscript within 60 days; if you cannot complete the modification within this time period, please contact me. If you do not wish to modify the manuscript and prefer to submit it to another journal, notify me immediately so that the manuscript may be formally withdrawn from consideration by Spectrum.

Revision Guidelines
To submit your modified manuscript, log into the submission site at https://spectrum.msubmit.net/cgi-bin/main.plex. Go to Author Tasks and click the appropriate manuscript title to begin. The information you entered when you first submitted the paper will be displayed; update this as necessary. Note the following requirements:

• Upload point-by-point responses to the issues raised by the reviewers in a file named "Response to Reviewers," NOT in your cover letter.
• Upload a compare copy of the manuscript (without figures) as a "Marked-Up Manuscript" file.
• Upload a clean .DOC/.DOCX version of the revised manuscript and remove the previous version.
• Each figure must be uploaded as a separate, editable, high-resolution file (TIFF or EPS preferred), and any multipanel figures must be assembled into one file.
• Any supplemental material intended for posting by ASM should be uploaded with their legends separate from the main manuscript. You can combine all supplemental material into one file (preferred) or split it into a maximum of 10 files with all associated legends included.

For complete guidelines on revision requirements, see our Submission and Review Process webpage. Submission of a paper that does not conform to guidelines may delay acceptance of your manuscript.

Data availability: ASM policy requires that data be available to the public upon online posting of the article, so please verify all links to sequence records, if present, and make sure that each number retrieves the full record of the data. If a new accession number is not linked or a link is broken, provide Spectrum production staff with the correct URL for the record. If the accession numbers for new data are not publicly accessible before the expected online posting of the article, publication may be delayed; please contact production staff (Spectrum@asmusa.org) immediately with the expected release date.

Publication Fees: For information on publication fees and which article types are subject to charges, visit our website. If your manuscript is accepted for publication and any fees apply, you will be contacted separately about payment during the production process; please follow the instructions in that e-mail. Arrangements for payment must be made before your article is published.

ASM Membership: Corresponding authors may join or renew ASM membership to obtain discounts on publication fees. Need to upgrade your membership level? Please contact Customer Service at Service@asmusa.org.

The ASM Journals program strives for constant improvement in our submission and publication process. Please tell us how we can improve your experience by taking this quick Author Survey.

Thank you for submitting your paper to Spectrum.

Sincerely,
Day-Yu Chao
Editor
Microbiology Spectrum

Response of reviewer comments

Thank you for the comments to the submitted version of this commentary. I have implimented all the suggested changes.

1. Line 24, ...sequencing can be clarified as "next-generation sequencing".

- It has been changed.

2. Line 82, "Field sampling can show where the virus is present" can be further clarified as "Field sampling with broad geographical coverage can show where the virus is present and avoid surveillance disparity in sampling efforts and sequencing capacity".

- Thanks, the change has been done.

3. Line 86, "Cell culture..." can be further clarified as "Virus culture in different tick or mammalian cell lines..."

- It has been clarified.

4. Line 97-101, I suggest consolidating these sentences, such as "The presence of JMTV RNA in ticks is not yet clinically relevant, but it is a strong reason to use the ensemble approach to evaluate the risk to public health and propose the One-Health approach."

- Thanks for the suggestion. Implimented.

Re: Spectrum02210-26R1 (Viral surveillance beyond detection: JMTV and the need for ensemble approaches in emerging virus discovery)

Dear Prof. Vibhu Prasad:

Your manuscript has been accepted, and I am forwarding it to the ASM production staff for publication. Your paper will first be checked to make sure all elements meet the technical requirements. ASM staff will contact you if anything needs to be revised before copyediting and production can begin. Otherwise, you will be notified when your proofs are ready to be viewed.

Data Availability: ASM policy requires that data be available to the public upon online posting of the article, so please verify all links to sequence records, if present, and make sure that each number retrieves the full record of the data. If a new accession number is not linked or a link is broken, provide production staff with the correct URL for the record. If the accession numbers for new data are not publicly accessible before the expected online posting of the article, publication may be delayed; please contact ASM production staff immediately with the expected release date.

Publication Fees: For information on publication fees and which article types have charges, please visit our website. We have partnered with Copyright Clearance Center (CCC) to collect author charges. If fees apply to your paper, you will receive a message from no-reply@copyright.com with further instructions. For questions related to paying charges through RightsLink, please contact CCC at ASM_Support@copyright.com or toll free at +1-877-622-5543. CCC makes every attempt to respond to all emails within 24 hours.

ASM Membership: Corresponding authors may join or renew ASM membership to obtain discounts on publication fees. Need to upgrade your membership level? Please contact Customer Service at Service@asmusa.org.

Embargo Policy: A press release may be issued as soon as the manuscript is posted on the Spectrum Latest Articles webpage. The corresponding author will receive an email with the subject line "ASM Journals Author Services Notification" when the article is available online.

Cover Image Submissions: If you would like to submit a potential Cover Image, please email a file and a short legend to spectrum@asmusa.org. Please note that we can only consider images that (i) the authors created or own and (ii) have not been previously published. By submitting, you agree that the image can be used under the same terms as the published article. Image File requirements: TIF/EPS, 7.5 inches wide by 8.25 inches tall (at least 2,250 pixels wide by 2,475 pixels tall), minimum 300 dpi resolution (600 dpi preferred), RGB, and no figure elements, e.g., arrows or panel labels. The legend should be a short description of the image, 1-2 sentences recommended. Please use the ASM Journals Cover Image Sizing Tool (https://journals.asm.org/pb-assets/html/asm-cover-sizing-tool.html) to ensure that your proposed cover image meets our size requirements.

The ASM Journals program strives for constant improvement in our submission and publication process. Please tell us how we can improve your experience by taking this quick Author Survey.

Thank you for submitting your paper to Spectrum.

Sincerely,
Day-Yu Chao
Editor
Microbiology Spectrum
